# The impact of rapid malaria diagnostic tests upon anti-malarial sales in community pharmacies in Gwagwalada, Nigeria

**DOI:** 10.1186/1475-2875-12-380

**Published:** 2013-10-30

**Authors:** John O Ikwuobe, Brian E Faragher, Gafar Alawode, David G Lalloo

**Affiliations:** 1 Department of Clinical Sciences, Liverpool School of Tropical Medicine, Liverpool, UK; 2 Partnership for Transforming Health Systems (PATH), Abuja, Nigeria

**Keywords:** Rapid diagnostic test (RDT), Anti-malarial, Malaria, Pharmacy

## Abstract

**Background:**

Rapid diagnostics tests for malaria (RDT) have become established as a practical solution to the challenges of parasitological confirmation of malaria before treatment in the public sector. However, little is known of their impact in private health sector facilities, such as pharmacies and drug shops. This study aimed to assess the incidence of malaria among unwell patients seeking anti-malarial treatment in two community pharmacies in Nigeria and measure the impact RDTs have on anti-malarial sales.

**Methods:**

This was a comparison study of two pharmacies located in the suburbs of Gwagwalada, in the Federal Capital Territory of Nigeria, between May and July 2012. In the intervention arm, patients seeking to purchase anti-malarials had an RDT performed before treatment while the control pharmacy continued normal routine practice.

**Results:**

A total of 1,226 participants were enrolled into the study. The incidence of malaria in the intervention arm (n = 619) was 13.6% and adolescent participants had a statistically significant higher incidence (26.0%) compared to adults (11.9%) (P = 0.001). A history of fever in the last 48 hours was associated with a statistically significant higher incidence of malaria (28.3%) (P < 0.001). Having a RDT test reduced the chance of purchasing an anti-malarial by 42% (95% CI: 38%-46%) compared to not having a test. 51.6% (276) of the study participants with a RDT negative result still purchased anti-malarials, especially if anti-malarials had been recommended by a health professional (58.9%) compared to self-referral (44.2%) (P = 0.001). Patients with RDT negative results were also more likely to purchase an anti-malarial if there was a reported malaria positive laboratory test prior to presentation (66.2%; P = 0.007), a history of fever in the last 48 hours (60.5%; P = 0.027), and primary school education or less (69.4%; P = 0.009). After adjusting for age group and gender differences, having at least a secondary school education reduced the chance of buying an anti-malarial (OR 0.504 (95% CI: 0.256-0.993)) compared to having primary education or lower.

**Conclusion:**

The study highlights the enormous potential for improving appropriate prescription of anti-malarials in pharmacies and preventing unnecessary use of artemisinin combination therapy (ACT).

## Background

Malaria has been a global public health problem for more than a century, claiming the lives of millions each year and reducing the quality of life of many others, especially in sub-Saharan Africa (SSA), where it still remains a serious concern [1]. The declining morbidity and mortality from malaria [2-10] raises concerns about the rationale for presumptive management of malaria. Despite its questionable economic value, presumptive management of malaria is still common in SSA. Many patients are inappropriately treated with anti-malarials with no improvement, exposure to unnecessary potential side effects and missed opportunities to treat the real cause of fever [11,12]. However, prompt parasitological confirmation of malaria before treatment is unrealistic in many settings because expert laboratory diagnostic services are scarce or unavailable. The provision of cheap and accurate rapid point of care tests, that require little skill, is therefore appealing. In 2011, the World Health Organization (WHO) expanded their guidelines for the management of uncomplicated malaria to specifically include the use of rapid diagnostic tests (RDTs) for confirmation of malaria before treatment. Despite this, the use of RDTs remains disappointingly low in public health sector facilities in most malaria endemic countries [1]. Several studies also show that RDT use itself may or may not influence a change in the anti-malarial prescription behaviour of doctors and other health workers [13-17], but little is known about RDT use in private health sector facilities - even though the private sector manages a substantial burden of presumed malaria in SSA.

The WHO Global Malaria initiative in March 2012 launched the “T3 initiative” to reaffirm the importance of RDTs in malaria control. This initiative includes: testing all suspected cases; treating these cases with ACT; and tracking (monitoring) malaria control programmes. It aims to help policy makers and donors recognize the importance of scaling up diagnostic testing services (especially RDT) to match the funding that is already available for ACT. Studies are needed in private health sector facilities such as pharmacies and drug shops, to build upon existing evidence that demonstrates the value of RDTs in malaria control. This study examined the impact of the introduction of RDTs upon sales of anti-malarials in a community pharmacy in Gwagwalada, Nigeria, to assess the incidence of malaria among adolescents and adults who are unwell and seeking treatment for malaria and to determine the factors that influenced prescription of anti-malarials when the malaria RDT was negative.

## Methods

### Study area

The study was conducted in the suburbs of Gwagwalada area council, one of six area councils in the Federal Capital Territory of Nigeria, a region reported to be holo-endemic and perennial for malaria as at the time of the study [18], but now regarded as meso-endemic in an unpublished report by the National Malaria Control Programme (NMCP) [19]. ITN coverage and use are reportedly low among the population and malaria accounts for 52% of all inpatient and outpatient visits. Although the council is covered by a wide variety of government and privately owned health facilities, the health worker to patient ratio is still very low (1: 11335) [20]. Health services at these facilities are not free and the national anti-malarial treatment policy of Nigeria does not exclude the sale of anti-malarials without a doctors’ prescription. Thus, anti-malarials are easily accessible in the free market, especially in pharmacies. The national malaria treatment policy, since 2004, includes the use of artemether-lumefantrine and artemether-amodiaquine as first and second line treatment of uncomplicated malaria respectively [21]. However, the use of anti-malarials as monotherapies is still common.

### Study design and sampling strategy

The study was a comparison between two pharmacies. Pharmacies with an average of at least 23 anti-malarial sales per day in the council area were identified from a list of eligible pharmacies obtained from the Association of Community Pharmacists of Nigeria. Pharmacies were approached to see if they would be willing to participate in the study. Two community pharmacies were chosen at random from the list of pharmacies willing to participate. An initial study was undertaken to determine the common symptoms of uncomplicated malaria that prompted patients who were unwell to seek anti-malarial treatment and to identify the routine prescription practice in both pharmacies, checking sales records to ensure that baseline average number of anti-malarial sales per day for both pharmacies were similar. The initial study also determined that anti-malarial prescription practice, the availability of different class of anti-malarials and their cost were similar in both pharmacies. The intervention pharmacy was then chosen at random and provided with RDTs: the control continued with its routine community pharmacy practice.

Participants were eligible if they had symptoms of uncomplicated malaria and were at least ten years of age or older. Patients were assessed by the study nurse and excluded from the study if they had symptoms of severe malaria or were pregnant. Severe malaria was considered as those with symptoms of malaria and pallor, extreme weakness and inability to walk, or an inability to tolerate oral medications. Female adult patients were excluded if they said they were pregnant, if their last menstrual period was more than 35 days prior to date of interview or if they could not remember the date of their last menstrual period.

In the intervention pharmacy, participants gave written consent after the study had been explained to them. Participants presenting to the intervention pharmacy (either with a malaria prescription or those seeking to buy anti-malarials for self-medication) with symptoms of uncomplicated malaria were tested using RDTs and shown the results before drugs were dispensed. The results were also revealed to the pharmacist. In the case of an RDT negative result, a decision to suspend anti-malarial treatment was made following further discussion between the pharmacist and the patient. The investigator was not involved in the decision.

An indicative sample size for the study was calculated assuming that the number of anti-malarial sales per day in both pharmacies (prior to the intervention) followed a statistical Poisson distribution with mean 23. Assuming 35 days of observation (during which time 1,610 patients were expected to participate) and no change in the sales rate in the control pharmacy, the study had 90% power to detect a reduction in the average daily number of anti-malarial sales in the intervention pharmacy from 23 to 19.2 (a 16% reduction).

Rapid tests were conducted using SD BIOLINE Malaria Antigen Pf (Lot Number: 082112, REF 05FK50, Manufacture date: 18-07-2011, Expiry date: 17-07-2013) by the study nurse. The kit is included in the WHO/Global Fund list of quality assured RDTs worldwide [22]. The tests were carried out in accordance to the instructions in the manufacturers’ manual. Standard universal precautions were observed. A positive result was indicated when there were two colour bands (Pf test line and C test line) within the result window. The presence of one colour band (C test line) and no colour band indicated a negative or an invalid result respectively. The test kit detects HRP-II antigen of *Plasmodium falciparum* and is reported to be very stable at temperatures up to 40°C. The sensitivity and specificity of the kit were reported by the manufacturers as 99.7% and 99.5% respectively. Quality assurance tests were carried out on the batch of kits used by the Liverpool School of Tropical Medicine (LSTM) laboratory by testing 25 samples of the RDTs with known positive and negative malaria samples (confirmed by expert microscopy), replicating field conditions as much as possible. The kits showed 100% sensitivity and specificity.

Data was collected using Epi-Info version 7 and analysed using SPSS version 20. The incidence of malaria was calculated for all participants and for specific sub groups of particular interest and presented as percentages. The risk factors for malaria were analysed by multivariate analysis first and then by multivariate linear regression analysis for statistical significance, adjusting for age group and gender differences in the population. The statistical significance level for this study was set at 5%. To determine the impact of RDTs on anti-malarial sales, a cross tabulation of anti-malarial sales between the two study arms was analysed for risk estimates with 95% Confidence Intervals (95% CI). The risk difference in sales was calculated by calculating risk ratios and testing it statistically with Chi-squared tests. To determine the effect of each variable on anti-malarial sale, a multivariate analysis comparing anti-malarial sales and each variable was tested for statistical significance using Chi-squared tests. All the variables that were statistically significant were then adjusted for age group and gender differences by including them into a multivariate linear regression model through a generalized linear model. The adjusted odds ratios obtained were interpreted with a 95% CI.

Ethical approval was granted by the National Health Research Ethics Committee of Nigeria (NHREC) and the LSTM Research Ethics Committee.

## Results

**Figure 1:**
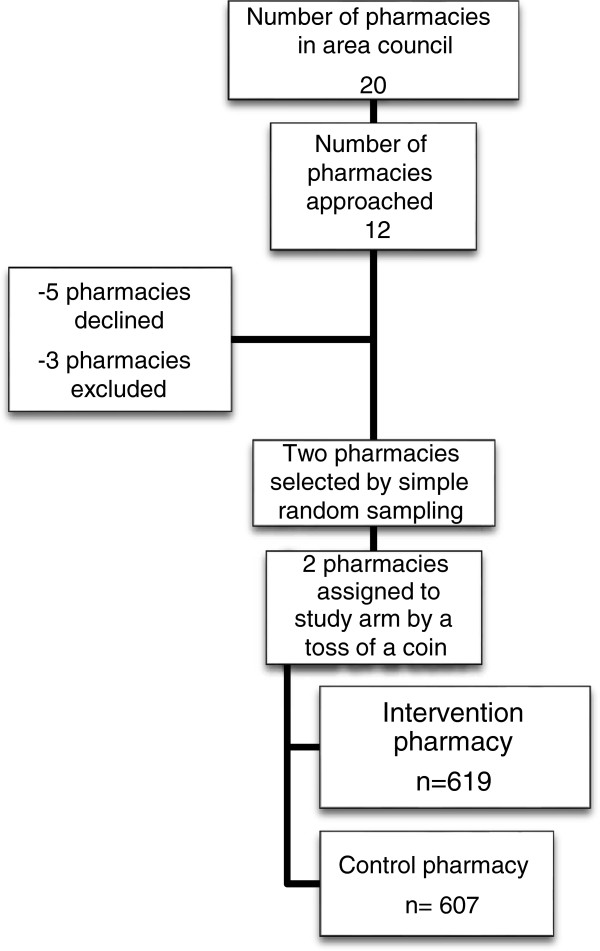
Schematic summary of the number and disposition of the pharmacies at each stage of selection.

12 of the 20 registered pharmacies in the area council were initially approached to participate in the study. Five pharmacies declined participation for fear of their records being published, while three pharmacies were excluded because they did not have sufficient daily anti-malarial sales to reach the intended sample size for the stipulated period of the study. Figure 1 shows a summary of the number and disposition of the pharmacies at each stage of selection.

The study took place between May and July 2012. A total of 1226 participants aged 10-78 years (mean age of 30.847, 51.7% females and 88.7% adults) were enrolled into the study. A large proportion of the study participants (90.4%) had at least a secondary education while 60.5% of the study population had a below average monthly income. A detailed summary of the baseline characteristics of participants in the study is shown in Table 1.

**Table 1:** Baseline characteristics of study participants

	**Control pharmacy**	**Intervention pharmacy**	**Total (n = 1226) frequency/mean difference (95% CI)**	**P value**
	**n = 607**	**n = 619**		
Gender				
-Female	328 (54.0%)	306 (49.4%)	634 (51.7%)	0.107
-Male	279 (46.0%)	313 (50.6%)	592 (48.3%)	
Age (years)	30.12 (10.23)	31.55 (11.46)	1.42 (0.20-2.64)	0.022
Age group				
-Adolescent	65 (10.7%)	73 (11.8%)	138 (11.3%)	0.548
-Adult	542 (89.3%)	546 (88.2%)	1088 (88.7%)	
Level of education (whole population)				
-Primary or less	61 (10%)	57 (9.2%)	118 (9.6%)	0.618
-At least secondary	546 (90%)	562 (90.8%)	1108 (90.4%)	
Level of income				
-Less than average income	372 (61.3%)	370 (60.5%)	742 (60.5%)	0.588
-Average income or more	235 (38.7%)	249 (40.2%)	484 (39.5%)	
Reported last treatment for malaria				
-Less than 6 months ago	381 (62.8%)	386 (62.4%)	767 (62.6%)	0.882
-More than 6 months ago	226 (37.2%)	233 (37.6%)	459 (37.4%)	
Who recommended anti-malarial				
-Self	255 (42.0%)	303 (48.9%)	558 (45.5%)	0.015
-Health professional	352 (58.0%)	316 (51.1%)	668 (54.5%)	
Participants with a doctor’s prescription prior to purchase of anti-malarial	169 (27.8%)	52 (8.4%))	221 (18.0%)	< 0.001
Reported positive lab test prior to purchase of anti-malarial	103 (17.0%)	96 (15.5%)	199 (16.2%)	0.488
History of fever in the last 48 hours	394 (64.9%)	166 (26.8%)	560 (45.7%)	< 0.001

### Incidence and risk factors for malaria

All 619 patients enrolled in the intervention arm were tested for malaria with RDTs and shown the results before an anti-malarial was dispensed. The incidence of malaria in the intervention arm was 13.6%. Adolescent participants had a statistically significant higher incidence (26.0%) compared to adults (11.9%) (P = 0.001). Table 2 summarizes the incidence of malaria in the different sub-study groups analysed. A history of fever in the last 48 hours (26.8% of the test population) was associated with a statistically significant higher incidence of malaria (28.3%) (P < 0.001) and 47.1% of adolescents with a history of fever in the last 48 hours had malaria. Only 22.9% of participants with a reported recent positive lab test for malaria at another clinic had malaria on RDT testing. There was no significant difference between the incidence of malaria in the sub-population of participants who either self-recommended malaria treatment (12.5%), or got a recommendation from a health professional (14.6%).

**Table 2:** Incidence of malaria (using RDTs) in different sub-populations of patients in the intervention arm

**Sub-population**	**Incidence (%)**	**Total number of participants tested within sub-population (% distribution within the total population)**
Gender		
Female	11.4	306 (49.0%)
Male	15.7	313 (51.0%)
Age group		
Adolescent	26.0	73 (11.8%)
Adult	11.9	546 (88.2%)
Level of education (adults)		
Primary education or less	11.8	34 (33.5%)
Secondary education or more	11.9	512 (66.5%)
Level of income (adults)		
Less than average	10.8	297 (59.8%)
Greater than average	13.3	249 (40.2%)
Who recommended anti-malarial?		
Self	12.5	303 (49.0%)
Health professional	14.6	316 (51.0%)

Table 3 shows the results of a multivariate regression model that examined risk factors for malaria in the study population. Adults were much less likely to have malaria than adolescents (OR 0.514 (95% CI: (0.280-0.946) and malaria was much more common if there was a history of fever in the last 48 hours (OR 4.027 (95% CI: 2.482-6.352)).

**Table 3:** Multivariate analysis examining risk factors for RDT confirmed malaria

**Risk factors for malaria**	**Odds ratio**	**Adjusted odds ratio**
	**(95% CI)**	**(95% CI)**
	**P value**	**P value**
Adult age group	0.384 (0.214-0.688)	0.514 (0.280-0.946)
	0.001	0.033
Reported positive lab test prior to presentation	2.221 (1.282-3.812)	2.177 (1.208-3.925)
	0.004	0.010
A history of fever in the last 48 hours	4.441 (2.757-7.151)	4.027 (2.482-6.532)
	< 0.001	< 0.001
Reported last treatment for malaria (more than 6 months ago)	1.611 (1.014-2.560)	1.627 (1.002-2.640)
	0.042	0.049

### Effect of RDT upon anti-malarial sales

**Figure 2:**
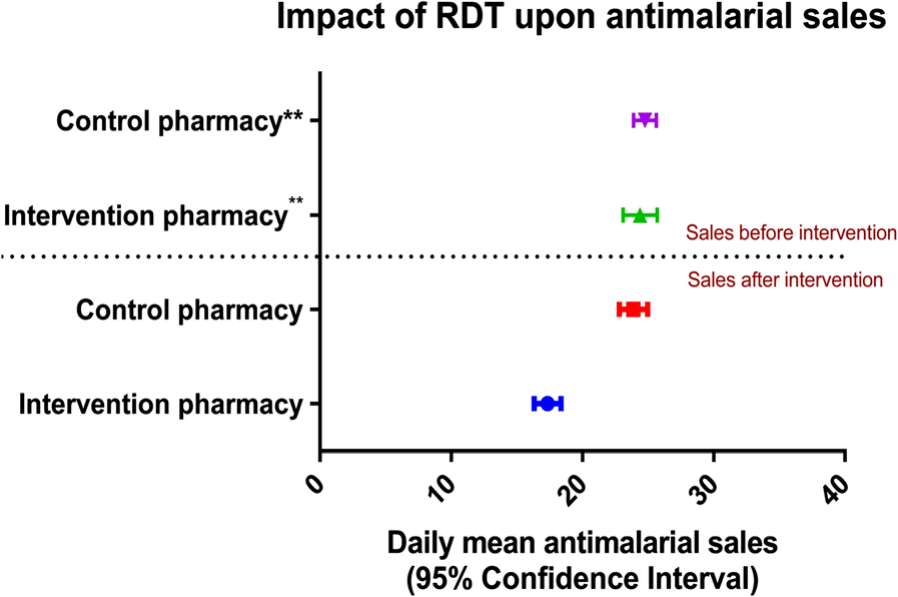
A box plot showing the total mean daily number of anti-malarial sales in the intervention and control pharmacy 35 days prior to the start of the study (Retrospective) and 35 days after the study commenced (Prospective).

An anti-malarial was only dispensed to 360 (59.2%) of the participants in the intervention pharmacy compared to 607 (100%) of the participants in the control arm. Having an RDT test reduced the chance of purchasing an anti-malarial by 42% (95% CI: 38%-46%) compared to not having a test. Figure 2 shows the daily anti-malarial sales for each pharmacy before and during the study. 51.6% (276) of the study participants with an RDT negative result still purchased anti-malarials. Patients were more inclined to purchase an anti-malarial, even though RDT result was negative, if it had been recommended by a health professional (58.9%) compared to self-referral (44.2%) (P = 0.001). Patients with RDT negative results were also more likely to purchase an anti-malarial if there was a reported malaria positive lab test prior to presentation (66.2%; P = 0.007), a history of fever in the last 48 hours (60.5%; P = 0.027), and primary school education or less (69.4%; P = 0.009).

**Figure 3:**
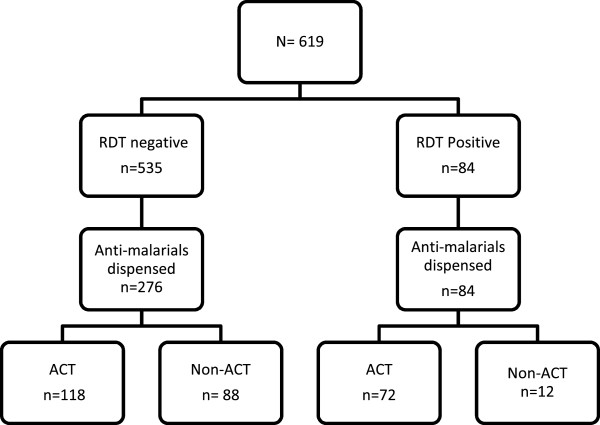
Schematic summary of the number and disposition of anti-malarials within different sub-groups in the intervention pharmacy.

A multivariate analysis (Table 4), showed that after adjusting for age group and gender differences, having at least a secondary school education reduced the chance of buying an anti-malarial (OR 0.504 (95% CI: 0.256-0.993)) compared to having primary education or lower. Having an anti-malarial recommended to the patient by a health professional and reporting a previous positive lab result prior to presentation significantly increased the chance of an anti-malarial sale. The prescription rates of different anti-malarial drugs and their disposition within both study arms and sub-groups in the intervention pharmacy are provided in Figure 3 and Table 5.

**Table 4:** Selected risk factors for anti-malarial purchase after an RDT negative test result showing adjusted and unadjusted odds

**Risk factors for anti-malarial sale**	**Odds ratio intervention (RDT negatives only) (95% CI) P value**	**Adjusted odds ratio (RDT negatives only) (95% CI) P value**
Average income or more	0.658 (0.465-0.931)	0.687 (0.468-1.007)
	0.018	0.054
At least secondary education	0.438 (0.232-0.824)	0.504 (0.256-0.993)
	0.009	0.048
Reported positive lab test prior to presentation	2.020 (1.207-3.382)	1.737 (1.007-2.996)
	0.007	0.047
Reported last treatment for malaria (more than 6 months ago)	0.782 (0.549-1.114)	0.714 (0.494-1.032)
	0.173	0.073
Anti-malarial recommended by a health professional	1.812 (1.286-2.553)	1.617 (1.134-2.305)
	0.001	0.008
A history of fever in the last 48 hours	1.592 (1.051-2.410)	1.440 (0.934-2.218)
	0.027	0.098

**Table 5:** Anti-malarial prescription practice in both study arms showing the frequency of use of different ACT and non-ACT drugs within each study group

**Antimalarial dispensed**	**Intervention pharmacy (frequency and % antimalarials within study arm) n = 360**	**Control pharmacy (frequency and % antimalarials within study arm) n = 607**
Artemether-Lumefantrine	180	337
	(50.0%)	(55.5%)
Artesunate-Amodiaquine	42	47
	(11.7%)	(7.7%)
Dihydroartemisinin-Piperaquine	30	33
	(8.3%)	(5.4%)
Artesunate-Sulfadoxine-Pyrimethamine	8	12
	(2.2%)	(2.0%)
ACT (total)	260	429
	(72.2%)	(70.6%)
Sulfadoxine-Pyrimethamine alone	39	75
	(10.8%)	(12.4%)
Artesunate alone	47	58
	(13.1%)	(9.6%)
Chloroquine alone	13	30
	(3.6%)	(4.9%)
Quinine alone	1	15
	(0.3%)	(2.5%)
Non-ACT (total)	100	178
	(27.8%)	(29.4%)

## Discussion

This study is the first of its kind in West Africa, and only the second study in Africa, that attempts to show the effect of RDTs on anti-malarial sales in pharmacies and it highlighted a number of interesting findings. An overall incidence of 13.6% was slightly lower than expected, considering that the study was conducted at a time when the study area was considered holo-endemic for malaria [18]; however it now is regarded as meso-endemic in an unpublished report by the NMCP [19]. The result is in line with other studies that demonstrate the relatively low proportions of fever caused by malaria. The incidence in adolescents was higher (26.0%) compared to the adult age group (11.0%), especially if they had a history of fever in the last 48 hours (47.1%). Epidemiologically, adolescents and adults tend to be classified together on the basis of incidence and clinical presentation of malaria even though it is well documented that the incidence of malaria reduces progressively with increasing age after the age of five [23]. The results again highlight the need to better understand the dynamics of malaria in this age group.

Studies have suggested the introduction of RDTs to pharmacies in recognition of its importance in the treatment of malaria in most African settings [24], but only one previous African study has demonstrated its effect on anti-malarial prescription [25]. This study clearly shows that having an RDT before treatment reduces the chance of selling anti-malarials to adolescents or non-pregnant adults with symptoms of uncomplicated malaria in a community pharmacy by 42%. The estimated reduction from this study is considerably less than that reported by other studies, which showed a 77% [26] and 96% [27] reduction in anti-malarial prescriptions with RDT use. However, there are good reasons for this difference. Firstly, unlike these studies, this study did not interfere with the decision of pharmacists or patients to treat malaria, irrespective of the outcome of the result, as this is likely to be the case in most pharmacies in Nigeria. Secondly, as opposed to government owned public health facilities, pharmacies are primarily established for profit. Therefore, it may prove extremely difficult to persuade pharmacies to restrict the sale of anti-malarials to only RDT positive patients, unless alternative sources of income are made available. Zikusooka et al. [28] suggested that restricting anti-malarial treatment to only RDT positive patients will save up to $2.12 per person, assuming that RDT and ACT were sold at government approved prices of $0.95 and $2.40 respectively, when 25% or less of the population test positive for malaria. Bearing in mind the current cost of ACT in the free market (ACT accounted for 72.2% of anti-malarial prescriptions in the intervention study arm), a 42% reduction in anti-malarial sales would lead to considerable economic benefit if the overall incidence is 13.6%.

Prior to the start of this study, the study nurse, pharmacists and staff of the intervention pharmacy were trained on how to use RDTs. Most RDT tests were conducted by the study nurse, who was provided by the study team on the request of the intervention pharmacy, to reduce the workload on the pharmacists and other staff. This is representative of most pharmacies and drugs shops in Nigeria who employ nurses or Community Health Extension Workers, to assess patients, administer intravenous medications and give basic first aid treatment when required.

Substantial interest in the value of RDTs as a tool for proper case detection and management of malaria by trained health care personnel in the public health sector is well documented [29]. This study suggests that there should be more effort at extending proper case detection and management to pharmacies and other private health sector facilities, leveraging on already existing health care workers, with particular benefits in adults and adolescents seeking to buy anti-malarials without a reported history of fever in the last 48 hours.

In this study, only a previous reported positive laboratory result, greater than secondary education and recommendation of an anti-malarial by a health professional were found to significantly affect the sale of anti-malarials when the RDT result is negative. There is a wealth of evidence that better education is associated with improved health and better health seeking behaviour and the findings from this study are in line with this. It is understandable why anti-malarial recommendations from a health professional would significantly increase anti-malarial use even if the RDT was negative and this highlights the importance of educating health professionals to use appropriate diagnostic tests. Possessing a positive laboratory test (usually microscopy) prior to presentation also significantly increased the sale of anti-malarials to RDT negative patients. This is not consistent with findings from another study in Zambia, which suggests that anti-malarials are less likely to be prescribed if RDTs are used rather than microscopy [30]. The Zambian study was performed in a hospital with trained health professionals that had little faith in their microscopy results. Since RDT was a new intervention in that study and it seem to work, the clinicians were more inclined to adhere to the RDT results compared to microscopy. In this study, both pharmacists and patients were more inclined to believe the results of the laboratory test, as it was considered to be superior to the RDTs. However, considering that only 22.9% of participants with a reported positive laboratory test just prior to presentation tested positive for malaria, and in view of the quality assured RDT results, it is fair to say that the quality of routine microscopy in this setting is poor. Education campaigns aimed at pharmacists and patients might help to publicize the advantages of RDT use before anti-malarial prescription in this setting.

There are a number of limitations to this study; it involved only two sites and the presence of the investigator may have altered the behaviour of both pharmacist and patients- towards the end of the study, there was some evidence that the pharmacist in the intervention pharmacy was becoming concerned about loss of sales. Nevertheless, the magnitude of the change in anti-malarial prescription when using RDTs compared to the control pharmacy is considerable.

The authors believe that the pharmacies used are representative of registered community pharmacies in the study area because the pre-study assessment of registered pharmacies showed similarities in their daily average anti-malarial prescription rates and practices; including prescribing anti-malarials without a doctor’s prescription. In addition, there was no observed disparity in social class or educational status among patients patronizing the pharmacies. Furthermore, prices of drugs in community pharmacies are regulated by the Association of Community Pharmacies of Nigeria (ACPN), hence, prices and availability of anti-malarials were observed to be similar. However, because the study area is in the Federal Capital Territory of Nigeria, which hosts the ACPN, it is plausible that unregistered community pharmacies exist in the area. The study highlights the enormous potential for improving appropriate prescription of anti-malarials in pharmacies and preventing unnecessary ACT use. Further research on replicating the findings in other settings, and exploring how pharmacists could be incentivized to use this approach is urgently needed.

## Competing interest

The authors declare that they have no competing interests.

## Authors’ contributions

JI and GA conceived the study, and coordinated the acquisition of data. JI, BF and DL designed the study and participated in analysis and interpretation of data. JI and DL helped to draft the manuscript. All authors read and approved the final manuscript.

## References

[B1] WHO Test. Treat. Track: scaling up diagnostic testing, treatment and surveillance for malaria 2012 Geneva: World Health Organization

[B2] Ceesay SJ Casals-Pascual C Erskine J Anya SE Duah NO Fulford AJ Sesay SS Abubakar I Dunyo S Sey O Palmer A Fofana M Corrah T Bojang KA Whittle HC Greenwood BM Conway DJ Changes in malaria indices between 1999 and 2007 in The Gambia: a retrospective analysis Lancet 2008 372 1545 1554 10.1016/S0140-6736(08)61654-2 18984187 PMC2607025

[B3] Sharp BL Kleinschmidt I Streat E Maharaj R Barnes KI Durrheim DN Ridl FC Morris N Seocharan I Kunene S Seven years of regional malaria control collaboration—Mozambique, South Africa, and Swaziland Am J Trop Med Hyg 2007 76 42 47 17255227 PMC3749812

[B4] Murray CJ Rosenfeld LC Lim SS Andrews KG Foreman KJ Haring D Fullman N Naghavi M Lozano R Lopez AD Global malaria mortality between 1980 and 2010: a systematic analysis Lancet 2012 379 413 431 10.1016/S0140-6736(12)60034-8 22305225

[B5] Bhattarai A Ali AS Kachur SP Mårtensson A Abbas AK Khatib R Al-Mafazy AW Ramsan M Rotllant G Gerstenmaier JF Molteni F Abdulla S Montgomery SM Kaneko A Björkman A Impact of artemisinin-based combination therapy and insecticide-treated nets on malaria burden in Zanzibar PLoS Med 2007 4 e309 10.1371/journal.pmed.0040309 17988171 PMC2062481

[B6] Guerra CA Gikandi PW Tatem AJ Noor AM Smith DL Hay SI Snow RW The limits and intensity of *Plasmodium falciparum* transmission: implications for malaria control and elimination worldwide PLoS Med 2008 5 e38 10.1371/journal.pmed.0050038 18303939 PMC2253602

[B7] Cibulskis RE Aregawi M Williams R Otten M Dye C Worldwide Incidence of Malaria in 2009: Estimates, Time Trends, and a Critique of Methods PLoS Med 2011 8 e1001142 10.1371/journal.pmed.1001142 22205883 PMC3243721

[B8] Otten M Aregawi M Were W Karema C Medin A Bekele W Jima D Gausi K Komatsu R Korenromp E Low-Beer D Grabowsky M Initial evidence of reduction of malaria cases and deaths in Rwanda and Ethiopia due to rapid scale-up of malaria prevention and treatment Malar J 2009 8 14 10.1186/1475-2875-8-14 19144183 PMC2653503

[B9] Aregawi MW Ali AS Al-Mafazy AW Molteni F Katikiti S Warsame M Njau RJ Komatsu R Korenromp E Hosseini M Low-Beer D Bjorkman A D'Alessandro U Coosemans M Otten M Reductions in malaria and anaemia case and death burden at hospitals following scale-up of malaria control in Zanzibar, 1999-2008 Malar J 2011 10 10 46 10.1186/1475-2875-10-10 21332989 PMC3050777

[B10] Mmbando BP Vestergaard LS Kitua AY Lemnge MM Theander TG Lusingu JP A progressive declining in the burden of malaria in north-eastern Tanzania Malar J 2010 9 216 10.1186/1475-2875-9-216 20650014 PMC2920289

[B11] Chandler C Jones C Boniface G Juma K Reyburn H Whitty CJ Guidelines and mindlines: why do clinical staff over-diagnose malaria in Tanzania? A qualitative study Malar J 2008 7 53 10.1186/1475-2875-7-53 18384669 PMC2323020

[B12] Hume JC Barnish G Mangal T Armázio L Streat E Bates I Household cost of malaria overdiagnosis in rural Mozambique Malar J 2008 7 33 10.1186/1475-2875-7-33 18282270 PMC2279141

[B13] Hawkes M Kain KC Advances in malaria diagnosis Expert Rev Anti Infect Ther 2007 5 485 495 10.1586/14787210.5.3.485 17547512

[B14] Ansah EK Narh-Bana S Epokor M Akanpigbiam S Quartey AA Gyapong J Whitty CJ Rapid testing for malaria in settings where microscopy is available and peripheral clinics where only presumptive treatment is available: a randomised controlled trial in Ghana BMJ 2010 340 c930 10.1136/bmj.c930 20207689 PMC2833239

[B15] Reyburn H Mbakilwa H Mwangi R Mwerinde O Olomi R Drakeley C Whitty CJ Rapid diagnostic tests compared with malaria microscopy for guiding outpatient treatment of febrile illness in Tanzania: randomised trial BMJ 2007 334 403 10.1136/bmj.39073.496829.AE 17259188 PMC1804187

[B16] Uzochukwu BS Onwujekwe E Ezuma NN Ezeoke OP Ajuba MO Sibeudu FT Improving rational treatment of malaria: perceptions and influence of RDTs on prescribing behaviour of health workers in southeast Nigeria PLoS One 2011 6 e14627 10.1371/journal.pone.0014627 21297938 PMC3031496

[B17] Skarbinski J Ouma PO Causer LM Kariuki SK Barnwell JW Alaii JA de Oliveira AM Zurovac D Larson BA Snow RW Rowe AK Laserson KF Akhwale WS Slutsker L Hamel MJ Effect of malaria rapid diagnostic tests on the management of uncomplicated malaria with artemether-lumefantrine in Kenya: a cluster randomized trial Am J Trop Med Hyg 2009 80 919 926 19478249

[B18] Federal Ministry of Health, Abuja, Nigeria Strategic Plan 2009-2013: A Road Map for Malaria Control in Nigeria 2008 Federal Secretariat, Phase 3, Central Business District, Abuja, Nigeria: Federal Ministry of Health

[B19] Programme NMC Nigeria: Report on technical workshop on the mapping of malaria intensity in Nigeria 2013 1st floor, abia House, Central Area, Abuja, Nigeria: National Malaria Control Programme

[B20] Federal Capital Teritory, Nigeria FCT Health Statistical Bulletin 2010 Plot 2, Kapital Street, Area 11, Garki. PMB 24, FCT Abuja, Nigeria: FCTA Health and Human Services

[B21] WHO World Malaria Report 2012 Avenue Appia 20, 1211 Geneva, Switzerland: World Health Organization

[B22] WHO List of WHO/Global Fund quality assured RDTs http://www.google.com.ng/url?sa=t&rct=j&q=&esrc=s&source=web&cd=1&ved=0CCoQFjAA&url=http%3A%2F%2Fwww.theglobalfund.org%2Fdocuments%2Fpsm%2FPSM_QADiagnostics_Malaria_list%2F&ei=G-l0UryjD4mGswbCqoGIBg&usg=AFQjCNFqHdN9QnN7cdFyg2ALTL6HzqOr6Q&bvm=bv.55819444,d.Yms

[B23] Lalloo DG Olukoya P Olliaro P Malaria in adolescence: burden of disease, consequences, and opportunities for intervention Lancet Infect Dis 2006 6 780 793 10.1016/S1473-3099(06)70655-7 17123898

[B24] Chandler CI Hall-Clifford R Asaph T Pascal M Clarke S Mboye AK Introducing malaria rapid diagnostic tests at registered drug shops in Uganda: Limitations of diagnostic testing in the reality of diagnosis Soc Sci Med 2011 72 937 944 10.1016/j.socscimed.2011.01.009 21349623 PMC4194310

[B25] Cohen JL Dickens WT Laxminarayan R, Macauley MK Adoption of over-the-counter Malaria Diagnostics in Africa: the role of subsidies, beliefs, externalities, and competition The Value of Information 2012 Netherlands: Springer 173 191

[B26] D'Acremont V Kahama-Maro J Swai N Mtasiwa D Genton B Lengeler C Reduction of anti-malarial consumption after rapid diagnostic tests implementation in Dar es Salaam: a before-after and cluster randomized controlled study Malar J 2011 10 107 10.1186/1475-2875-10-107 21529365 PMC3108934

[B27] Msellem MI Mårtensson A Rotllant G Bhattarai A Strömberg J Kahigwa E Garcia M Petzold M Olumese P Ali A Björkman A Influence of rapid malaria diagnostic tests on treatment and health outcome in fever patients, Zanzibar—A crossover validation study PLoS Med 2009 6 e1000070 10.1371/journal.pmed.1000070 19399156 PMC2667629

[B28] Zikusooka CM McIntyre D Barnes KI Should countries implementing an artemisinin-based combination malaria treatment policy also introduce rapid diagnostic tests Malar J 2008 7 176 10.1186/1475-2875-7-176 18793410 PMC2556342

[B29] Thiam S Thior M Faye B Ndiop M Diouf ML Diouf MB Diallo I Fall FB Ndiaye JL Albertini A Lee E Jorgensen P Gaye O Bell D Major reduction in anti-malarial drug consumption in Senegal after nation-wide introduction of malaria rapid diagnostic tests PLoS One 2011 6 1 7 10.1371/journal.pone.0018419 PMC3071817 21494674

[B30] Hamer DH Ndhlovu M Zurovac D Fox M Yeboah-Antwi K Chanda P Sipilinyambe N Simon JL Snow RW Improved diagnostic testing and malaria treatment practices in Zambia JAMA 2007 297 2227 2231 10.1001/jama.297.20.2227 17519412 PMC2674546

